# A Systematic Review Exploring the Phytochemical Composition and Anticancer Activities of *Acacia catechu*

**DOI:** 10.3390/medsci13030161

**Published:** 2025-09-01

**Authors:** Navya Rana, Madhu Bala, Vinod Kumar, Rohitash Yadav, Neeraj Jain, Don Mathew, Khushboo Bisht, Rakesh Kumar, Sunil Kumar

**Affiliations:** 1 Department of Animal Sciences, Central University of Himachal Pradesh, Dharamshala 176206, Himachal Pradesh, India; navyarana98@gmail.com (N.R.); drthakurcuhp@gmail.com (R.K.); 2 Department of Pharmacology, Kalyan Singh Government Medical College, Bulandshahr 203002, Uttar Pradesh, India; drvinodaiimsrsk@gmail.com; 3 Department of Pharmacology, Pacific Medical College and Hospital, Udaipur 313001, Rajasthan, India; rohitashyadav1@gmail.com; 4 Department of Cancer Biology, CSIR CDIR, Lucknow 226031, Uttar Pradesh, India; neeraj.jainfellow@cdri.res.in; 5 Department of Biochemistry, Pacific Medical College and Hospital, Udaipur 313001, Rajasthan, India; mathewdon2@gmail.com; 6 Department of Pharmacology, All India Institute of Medical Sciences, Nagpur 441108, Maharashtra, India; khushboobisht12@gmail.com

**Keywords:** *Acacia catechu*, cancer, phytoconstituents, cell signaling pathways

## Abstract

**Background: ***Acacia catechu* is an important traditional medicinal plant that has been used to manage several ailments. Many in vitro and in vivo studies have demonstrated that it exhibits chemopreventive and antineoplastic effects by modulating diverse signaling pathways and molecular targets involved in cancer progression. This review attempts to systematically investigate the anticancer mechanisms of *A. catechu*, encompassing antiapoptotic, antioxidant, and antiproliferative activities. **Material and Methods:** This review was conducted using scientific databases such as Scopus, Web of Science, and Google Scholar, covering the studies from 2000 to 2024. The PRISMA methodology was applied, using the keywords *A. catechu*, phytoconstituents, and cancer. **Results:** A total of 39 studies were compiled from various databases that cited the biological use of *A. catechu*. The plant has an abundance of phenolic compounds, including catechin, epicatechin, epigallocatechin-3-O-gallate, and epicatechin-3-O-gallate, which show strong anticancer activities. The anticancer potential of *A. catechu* is explained as it regulates several modulators like reactive oxygen species and cytokines, and downregulates oncogenic molecules like c-myc and various signaling pathways, such as c-Jun and NF-κB. **Conclusions:** Our findings suggest that *A. catechu* and its bioactive constituents have the potential for cancer prevention and therapy. However, further mechanistic investigations using pure compounds, along with preclinical and clinical trials, are essential to translate this potential into clinical applications.

## 1. Introduction

**Figure 1:**
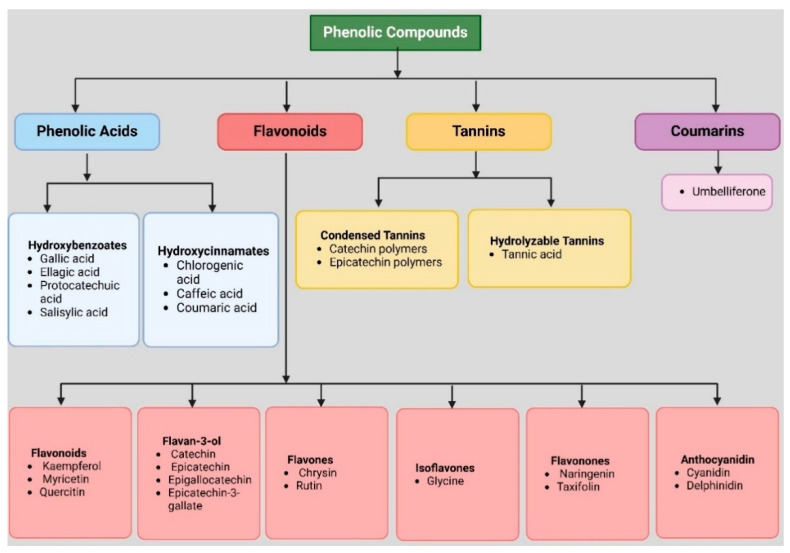
Phenolic compound classes of phytoconstituents in *A. catechu*.

Cancer is an uncontrollable cellular growth capable of invading adjacent cells or tissues and spreading [1]. In the year 2022, there were more than 20 million new cases of cancer and 9.7 million cancer-related deaths. It was estimated that one in five men and women would develop cancer during their lifetimes, while one in nine men and one in twelve women would die from it [2]. Current treatment approaches, such as chemotherapies and radiotherapies, have several adverse effects and are limited. Chemotherapy drugs, in particular, cause substantial damage to normal cells, making treatment painful. Patients experience various complications that ultimately have a poor impact on their quality of life [3]. In light of the increasing resistance to chemotherapies and radiotherapies, alternative treatments that are less harmful are being explored [4]. Plant-based drugs have gained much attention over the past few decades in the search for newer anticancer mechanisms. Cancer progression is often driven by chronic inflammation, oxidative stress, and dysregulated apoptotic pathways, making these biological processes key therapeutic targets. Natural compounds that modulate these mechanisms are being extensively studied for their potential to suppress tumor development [5]. *Acacia catechu,* commonly known as khair, catechu, and cutch, grows at an altitude of 1200 m in the sub-Himalayan tract of India, Pakistan, Bhutan, Nepal, Thailand, and China [6]. Several plant-derived phytochemicals have shown the ability to induce apoptosis, arrest the cell cycle, and inhibit angiogenesis in various cancer models. Given its rich phytoconstituent profile, *A. catechu* may exert many medicinal properties, including anti-inflammatory, antioxidant, antifungal, antibacterial, astringent, analgesic, anthelmintic, wound healing, antitumor, immune boosters, and antidiabetics [7]. *A. catechu* is an important bioactive plant and has been used traditionally to cure various diseases. In India, katha and cutch are two commercially important products made from the heartwood of *A. catechu*, which are used for various medical purposes [8]. Its bark has been used to treat cough, diarrhea, cold, sore throat, toothache, skin problems, and inflammatory conditions [9,10,11]. The stem, wood, and extracts are employed to relieve intestinal pain, fever, and mouth ulcers [12,13]. Parts of *A. catechu* are rich in phenolic compounds like catechin, epicatechin, gallic acid, and tannins (Figure 1) [7]. The phenolic compounds, more specifically, catechin (3,3′,4′,5,7-pentahydroxyflavan), are abundant in this plant and show anticancer, antioxidant, antiapoptotic, and anti-inflammatory properties [9,14]. There have been a few reviews published on *A. catechu*’s pharmacological activities [7,15], but none have examined its potential as a cancer therapy or chemopreventive agent in details. Consequently, *A. catechu* and its related anticancer capabilities and associated molecular targets have not been discussed. The present review aims to critically synthesize current evidences on the anticancer potential of *A. catechu*, focusing on its anti-apoptotic, antioxidant, and antiproliferative effects at cellular and molecular levels.

### Methodology

Using a systematic review methodology, this study aimed to answer the question, What are the anticancer properties of *A. catechu*? The following were the study’s objectives: (i) To classify diverse phytoconstituents in various parts of *A. catechu*; (ii) To examine pertinent research and review articles regarding *A. catechu’s* antiapoptotic, antioxidant, and antiproliferative activities; (iii) To provide key conclusions and recommendations for more therapeutic research.

Numerous significant scientific databases, such as “Scopus, Web of Science, and Google Scholar,” were searched for this work. The search technique contained pertinent terms like *Acacia catechu*, AND phytoconstituents, AND cancer. What was and was not included was decided by the authors. This systematic review includes studies published from 2000 to 2024.

Research not specifically about *A. catechu* and its involvement in cancer, as well as articles written in languages other than English (unless an English translation was available), were not included. Articles with relevant details were included, while those that did not meet the requirements were excluded. Numerous important topics were examined, such as phytoconstituents of *A. catechu* and its role in cancer. PRISMA (Preferred Reporting Items for Systematic Reviews and Meta-Analyses) was used to assess the included studies.

These approaches include the following steps:

**Identification:** The first stage in this process was to identify the research gap. The literature was then examined in context with the subject, and 743 records were found.

**Screening and eligibility:** This systematic review included in vitro and in vivo studies with the following inclusion criteria: (i) research detailing the phytochemical composition of *A. catechu and their role in cancer*; (ii) studies focusing specifically on *A. catechu* and its role in cancer prevention or treatment; (iii) articles discussing antioxidant, antiproliferative, and/or antiapoptotic mechanisms; (iv) studies written in English or with an available English translation.

**Figure 2:**
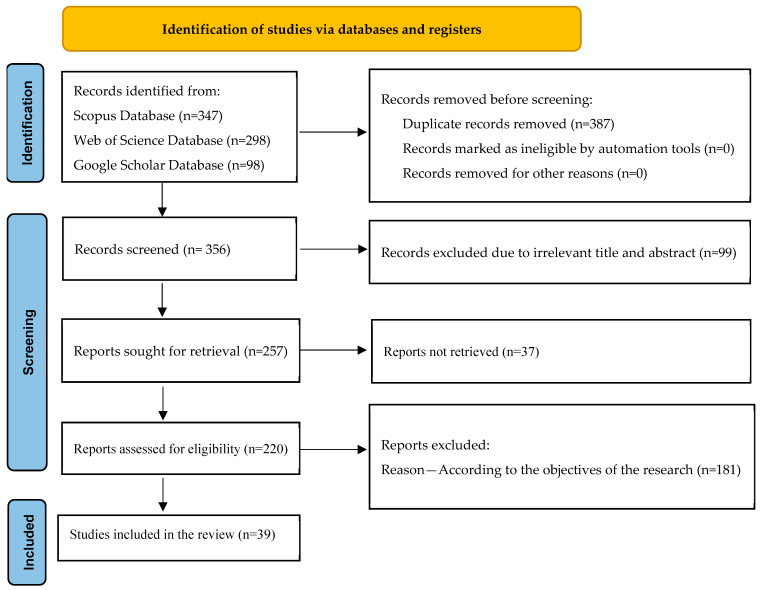
Systematic methodology of data collection from different databases.

**Exclusion criteria:** (i) Studies not directly investigating *A. catechu* in the context of cancer mechanisms; (ii) articles published in languages other than English without translation; (iii) case reports, conference abstracts, editorials, and commentaries. A total of 181 studies were excluded, and all the evaluated data from 39 eligible studies were compiled in the form of a review article (Figure 2).

**Data extraction and quality Assessment:** Relevant data were systematically extracted from all eligible studies. The extracted information included study identification (first author’s name and year of publication), study type and design (in vitro, in vivo, or review-based), experimental model (cell lines or animal models used), study objectives and outcomes, plant part used (e.g., heartwood, bark, leaves), type of extract or isolated phytoconstituents, phytochemical profile, concentration/dosage and treatment duration, observed biological activities, with emphasis on antiapoptotic, antioxidant, and antiproliferative effects, proposed mechanisms of action, and any toxicity or cytotoxicity data.

## 2. Role of Phytoconstituents of *A. catechu* in Cancer

The stem, bark, leaves, roots, and heartwood of *A. catechu* contain several active constituents that make it an effective medicinal herb in cancer treatment. Major phytochemical components are catechin, poriferasterol, epigallocatechin, epigallocatechin gallate, catechin, epicatechin, protocatechuic acid, quercetin, lupenone, kaempferol, L-arabinose, D-galactose, and afzelchin gum [16,17]. Table 1 lists the different phytoconstituents present in different parts of *A. catechu* with reported anticancer properties.

## 3. Cancer Pathways Modulated by *A. catechu*

### 3.1. Antiapoptotic Properties of A. catechu

Drugs derived from natural products inhibit angiogenesis and metastasis and promote apoptosis, three of the most significant characteristics of cancer. Apoptosis is the process of regulating cell death during organ development or maintaining cellular homeostasis by eliminating damaged or unnecessary cells [23]. *A. catechu* has shown strong proapoptotic activity via the intrinsic pathway. In MCF-7 breast cancer cells, the methanolic heartwood extract induced apoptosis by activating the caspase cascade and increasing the Bax/Bcl-2 ratio [24]. Chemopreventive effects were also observed in DMBA-induced mammary carcinoma in Balb/c mice treated with *A. catechu* heartwood extract, which led to normalized ductal morphology, chromatin condensation, cell shrinkage, breakdown of cells, and downregulation of oncogenes (p65 and c-Jun) and tumor suppressor gene p53 [25]. In SCC-25 epithelial cells, ethanolic seed extract upregulated intrinsic markers, including Bax, cytochrome c, and caspase-9, caspase-8, while downregulating Bcl-2 [26]. Similarly, in HT-29 colon cancer cells, heartwood extract induced ROS generation, loss of mitochondrial membrane potential (MMP), and activation of caspase 9 and 3, all hallmark events of intrinsic apoptosis [27]. Additionally, COLO-205 and HeLa cells treated with methanol and hexane bark extracts showed increased DNA fragmentation and apoptotic cell death, suggesting intrinsic pathway involvement, although the specific markers were not detailed [28].

### 3.2. Antioxidant Properties of A. catechu in Oncoprotection

The imbalance between prooxidants and antioxidants disrupts redox homeostasis, resulting in oxidative stress and excessive production of reactive oxygen species (ROS) [29]. This oxidative milieu not only initiates but also promotes cancer progression by inducing DNA damage, thereby causing genomic instability and replication errors. Elevated ROS further activates oncogenes and stress-responsive signal transduction pathways, amplifying tumorigenic signaling [30]. Multiple studies have highlighted the potent antioxidant and anticancer properties of *A. catechu* extracts. A study by Sasikala et al. (2022) showed that among six methanolic fractions of *A. catechu* stem bark, fraction 3 demonstrated the highest radical scavenging activity, 96.11% DPPH and 83% nitric oxide scavenging—correlating with a pronounced antiproliferative effect against MCF-7 cells (IC_50_ = 49.86 µg/mL). The study attributed this cytotoxicity to its capacity to attenuate oxidative stress–driven cancer cell proliferation [31]. Similarly, the aqueous bark extract exhibited strong antioxidant potential, as evidenced by DPPH (334.06 μg/mL), ABTS (259.35 μg/mL), FRAP (529.30 μg/mL), and CUPRAC (417.49 μg/mL) assays. This was associated with cytotoxicity against MCF-7 cells (IC_50_ = 137.5 μg/mL), further supporting an oxidative stress-mediated antiproliferative mechanism [32]. In colon cancer models, *A. catechu* extract displayed 90.82% DPPH radical scavenging at 100 μg/mL and induced ROS-mediated apoptosis in HT-29 cells (IC_50_ = 46.4 μg/mL) by disrupting mitochondrial membrane potential and activating the intrinsic apoptotic pathway. These findings emphasize its dual redox-modulating role, functioning as an antioxidant in normal cells while exerting prooxidant cytotoxic effects in cancer cells [33]. Guleria et al. (2011) further demonstrated the strong free radical–scavenging and DNA-protective potential of *A. catechu* heartwood. The ethyl acetate fraction exhibited low EC_50_ (half maximal effective concentration) values in DPPH (4.76 μg/mL), superoxide (26.21 μg/mL), and hydroxyl radical (33.69 μg/mL) assays, along with the highest reducing power. Moreover, ethyl acetate and acetone fractions effectively protected plasmid DNA from hydroxyl radical–induced strand breaks [34]. In an in vivo study, Monga et al. (2012) evaluated (+)-catechin–rich *A. catechu* heartwood extract (AQCE) against MCF-7 cells and DMBA-induced mammary carcinoma in Balb/c mice. AQCE significantly restored tumor marker profiles and enhanced antioxidant enzyme activities—including catalase, superoxide dismutase, glutathione peroxidase, glutathione reductase, glutathione-S-transferase, and thiol levels in mammary tissue and hepatic mitochondria. These effects underscored its ability to modulate both tumor biology and systemic antioxidant defense mechanisms [35]. Taken together, these studies provide compelling evidence that *A. catechu* exerts a multifaceted anticancer effect by modulating redox homeostasis. Its bioactive fractions rich in phenolic compounds, such as (+)-catechin, demonstrate potent radical scavenging abilities that protect normal cells from oxidative insult while paradoxically inducing lethal ROS accumulation in cancer cells. This dual redox modulation, coupled with its capacity to restore antioxidant enzyme systems and inhibit tumor proliferation in both in vitro and in vivo models, underscores its therapeutic promise. The convergence of antioxidant potency, DNA protective properties, and targeted prooxidant cytotoxicity positions *A. catechu* as a valuable candidate for integrative cancer management and as a potential lead for the development of redox-based anticancer therapeutics.

### 3.3. Antiproliferative Activities

**Figure 3:**
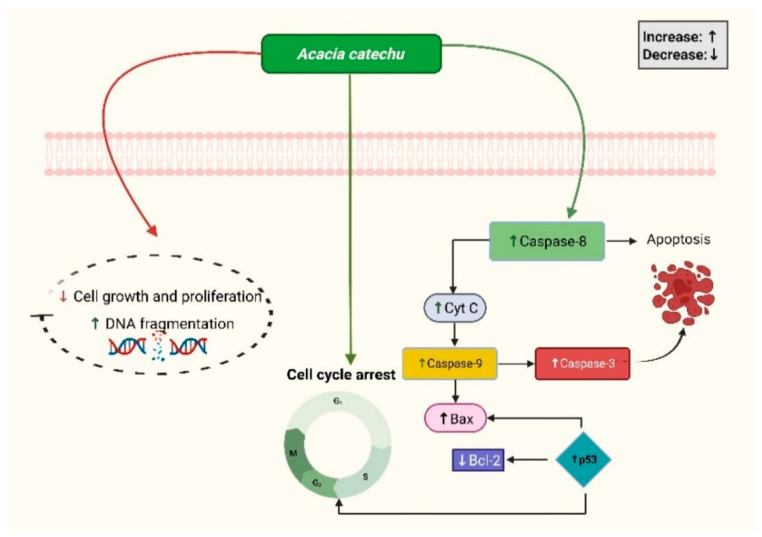
*A. catechu* anticancer properties based on molecular pathways. It suppresses cancer cell growth by inducing DNA fragmentation and cell cycle arrest. It activates apoptotic signaling through Bax, cytochrome c, caspase 3, 8, and 9, and p53, while downregulating Bcl-2, ultimately promoting apoptosis.

Plants with antiproliferative properties are critical in cancer treatment because they prevent cancer cells from rapidly multiplying. These plant-derived compounds target specific cellular pathways to inhibit or stop tumor growth (Figure 3). Methanolic extract of *A. catechu* heartwood (70% ACME) showed anticancer properties with an IC50 value of 105.35 μg [36]. *A. catechu* bark extract was tested in the MCF-7 cell line and demonstrated excellent anticancer activity and significantly reduced cell activity with an IC50 value of 49.86 µg/mL [31]. *A. catechu* was tested against brain (IMR-32), prostate (PC-3), liver (Hep-G2), breast (MCF-7), cervix (HeLa), and lung (A549) cell lines. Out of all cell lines, *A. catechu* was found to be most effective against MCF-7 cells with an IC50 of 137.5 µg/mL [32]. All concentrations of catechin hydrate and epigallocatechin from *A. catechu* spray-dried extract displayed antiproliferative activity of up to 20% with a slight decrease in lymphocyte viability [22]. In an in vivo study, tumor incidence was 100% in DMBA-treated mice, whereas in the catechin-rich extract-treated group, tumor incidence was significantly reduced by 62.5%. Further, in DMBA-treated animals, levels of NF-kB, p53, p65, and C-Jun were significantly increased, whereas heartwood extract significantly reduced C-Jun levels [25]. A study evaluated the antitumor activity of aqueous extracts of *A. catechu* heartwood (AQCE) against Balb/c mice induced with DMBA/TPA. Findings revealed a decrease in tumor volume and levels of MDA, GSH, and SOD, which indicates its chemopreventive activity [37]. The methanolic fruit extract of *A. catechu* was tested on two breast cancer cell lines, T47D and MCF-7. This extract showed high efficacy against the MCF-7 cell line (IC50 = 22.8 ± 4.9 μg/mL) and moderate efficacy against T47D cells (IC50 = 38.5 ± 1.4 μg/mL). Further treatment with *A. catechu* extract at a concentration of 100 µg/mL resulted in G2/M phase cell cycle arrest. Additionally, DNA fragmentation analysis in HL-60 cells revealed a necrotic smear pattern upon agarose gel electrophoresis following 24 h of incubation with the extract, indicating induction of necrotic cell death, suggesting that it may be used to treat cancer [38]. The efficacy of catechu, an active phytoconstituent of *A. catechu,* was tested on the MCF-7 cell line for cancer cell viability. *A. catechu’s* acetone: water extract at a concentration of 7:3 significantly inhibited cancer cell proliferation with an IC50 value of 0.5 μg/mL [39]. Table 2 discusses *A. catechu’s* anticancer properties and its related mechanisms.

## 4. Safety Profile

The safety of *A. catechu* was demonstrated by Chiano et al. (2020), where its extract showed significant cytotoxicity against HT-29 colon cancer cells, while not affecting the viability or contractile functionality of healthy rat ileum and proximal colon rings, even at concentrations up to 1000 µg/mL. This indicates a favorable safety profile and selective action toward cancerous cells, supporting its therapeutic potential without harming normal gastrointestinal tissues [27]. In another study, methanol and hexane extracts of *A. catechu* bark showed significant anticancer activity against COLO-205 and HeLa cell lines, while exhibiting minimal cytotoxicity toward human peripheral lymphocytes. This indicates a selective and safe profile, with potential applicability in animal and human systems [28].

## 5. Conclusions

This review compiled evidences demonstrating that *A. catechu* harbors various bioactive compounds with significant anticancer potential, attributed to its antioxidant, apoptotic, and antiproliferative properties. These multifaceted activities position *A. catechu* as a promising candidate for further investigation and potential integration into oncological therapies. Notably, the crude extract of *A. catechu* exhibits proapoptotic effects by upregulating the expression of pro-apoptotic Bax genes while concurrently downregulating the antiapoptotic Bcl-2 gene. This modulation leads to an increased Bax/Bcl-2 ratio, triggering cell shrinkage, the activation of caspase cascades, and the release of cytochrome c, collectively promoting apoptosis in cancer cells. Its remarkable antioxidant activity further highlights its anticancer potential. Its antiproliferative properties are evident through enhanced p53 expression and decreased tumor volume. Compiled and analyzed data over two decades of research, this review forms a basis for further exploration into synergistic combination approaches using *A. catechu* with other therapies to bring breakthroughs in integrative and holistic forms of cancer treatment.

## 6. Future Prospectives

Phytochemicals hold significant potential as anticancer drugs, and *A. catechu* harbors a variety of bioactive compounds. However, studies investigating isolated phytoconstituents from *A. catechu* and their specific mechanisms of action on animal models and cell lines are lacking. This gap could be filled by isolating phytochemicals and evaluating their anticancer properties in vitro and in vivo. Further, most of the studies are focused on plant extracts tested on cell lines, with limited data from rat/mouse models and no clinical trials. This highlights a significant gap for future research to explore its potential in clinical studies and anticancer drug development. Future studies in this field would improve the exploration of anticancer drugs’ mechanisms of action and aid in the discovery of safe and efficient treatments for cancer.

## Figures and Tables

**Table 1:** Distribution of major phytoconstituents in various parts of *A. catechu*.

Plant Part	Phytoconstituents	Cancer Mechanisms	References
Heartwood	Catechin	↓ Tumor growth, ↑ Antioxidant defense, ↓ c-jun levels	[18,19,20]
Leaves	Catechin, Epicatechin, Epigallocatechin-3-O-gallate, Epicatechin-3-O-gallate.	Antiproliferative activity	[21,22]

Note: ↑ indicates increase, ↓ indicates decrease.

**Table 2:** Potential antitumor properties and related mechanisms of action of *A. catechu.*

Cancer Type	Cell Line/Model	Plant Part	Concentration	Anticancer Effects	Mechanisms	References
Breast	MCF-7	Stem bark	7.8–1000 µg/mL	↑ Cytotoxicity	Not reported	[31]
Breast	MCF-7	Heartwood	1 ng–100 µg	↓ Cell proliferation, ↑ Cell cycle arrest, ↑ Apoptosis	Not reported	[36]
Colon	HT-29	Heartwood	0–1000 µg/mL	↓ Cell viability, ↑ Cytotoxicity, ↑ Apoptosis	↑ ROS, ↓ MMP, ↑ Caspase 3,9	[27]
Breast	MCF-7	Bark	50–250 µg/mL	DNA protection, Antiproliferative agent	-	[32]
Skin	SCC-25	Seeds	0.1–1000 µg/mL	↑ Cytotoxicity, ↑ Apoptosis	↑ Caspase 8,9, cytochrome C, Bax, ↓ Bcl-2	[26]
Leukemia	K562	-	10–100 µg/mL	↑ DNA damage, ↑ Cytotoxicity	↑ G2/M arrest	[38]
Breast	MCF-7/ Female Balb/c mice	Heartwood	10–100 µg/mL 50 mg/kg	↑ DNA fragmentation, ↑ Cell growth inhibition	↓ p53, c-jun, NF-κB	[25]
Breast	A431/ Balb/c mice	Heartwood	10–100 µg/mL 400 mg/kg	↓ Cell proliferation, visible necrotic keratinocytes, ↓ tumor burden	↑ LDH ↑ LPO, CAT, ↓ SOD, GSH, MDA	[37]
Breast	MCF-7	Heartwood	40–120 µg/mL	↑ Cell inhibition, inhibited animal fatty acid synthase	-	[39]

Note: ↑ indicates increase, ↓ indicates decrease.

## Data Availability

Not applicable.
